# The clinical performance of monoshade resin composite as posterior restoration: a randomized controlled clinical trial

**DOI:** 10.1038/s41598-025-08857-8

**Published:** 2025-07-12

**Authors:** Ruba Salah Anwar, Yasser Fathi Hussein, Mona Riad

**Affiliations:** 1 Conservative Dentistry Department, Faculty of Dentistry, Minia University, Minya, Egypt; 2 Dental Materials Department, Faculty of Dentistry, Minia University, Minya, Egypt; 3 Conservative Dentistry Department, Faculty of Dentistry, Cairo University, 11 El-Saraya St, Manial, Giza, Cairo Egypt

**Keywords:** Resin composite with a chameleon effect, Polyshade nanohybrid resin composite, Modified USPHS, Color match and stability, Health care, Materials science

## Abstract

The aim of this study was to assess the clinical performance of a monoshade universal resin composite as posterior restoration. Twenty adult patients having at least two carious lesions related to posterior teeth were selected. Each patient was provided with a monoshade resin composite (Omnichroma) and polyshade nanohybrid resin composite (Tetric^®^ N-Ceram) for class I or II restorations. The performance of these restorations was assessed at 1, 3, 6, 9, and 12 months according to the modified United States Public Health Service (USPHS) criteria. Statistical analysis was carried out employing the Friedmann test and the Wilcoxon signed-rank test, with a significance level of *p* = 0.05. None of the restorations exhibited any clinical conditions warranting replacement. The monoshade universal group revealed prevalence of (Bravo) scores concerning the anatomic form, surface texture and post-operative sensitivity through the follow-up period (*P* = 0.317). For color match and color stability, the polyshade group revealed a statistically significantly higher incidence of (Alpha) scores after 9 and 12 months (*P* = 0.025). After 12-months of follow-up, the monoshade universal resin composite demonstrated satisfactory clinical performance. It can serve as a viable substitute for polyshade nanohybrid composite where chair side time is an essential concern.

## Background

Replicating every natural characteristic of a tooth artificially is not always simple, as dentin and enamel differ in thickness, structure, composition, and, most notably, optical properties. Using a polyshade (polychromatic) or three-dimensional layering approach is a successful way to create direct resin composite (RC) restorations that look as natural as possible. With this method, the dentist can adjust the opacity and translucency of each layer to blend seamlessly with the neighboring teeth. Although the layering process requires more time and skill from the operator, it produces satisfactory outcomes in color matching^1,2^.

The goal of reducing the duration of restoration procedures and structuring color matching prompted dental manufacturers to compete in creating a universal RC (monoshade) that could be matched to a broad spectrum of universal tints. As the desire for enhanced esthetics and functionality continues to rise, the direction of RCs is shifting towards the integration of all-in-one technology. Clinicians are actively seeking materials that are user-friendly, time-efficient, and capable of meeting patients’ heightened expectations. Although no single material possesses all the necessary qualities, monoshade structurally colored universal RC may be paving the way for a future that offers sustainability, in which clinicians and patients no longer need to concern themselves with shade selection or replacing the fillings due to the failure of color matching^3,4^.

Tokuyama Dental America introduced the Smart Chromatic Technology of Omnichroma^®^ as the first manufactured monoshade universal RC, which enables the material to achieve extensive color-matching capabilities by producing a red-to-yellow structural color, similar to that of natural teeth. This chameleon effect is achieved by managing the size of the filler particles. The chameleon effect refers to a cosmetic characteristic that allows restorative material to blend with and mimic the color of its environment relying on the fact that resin composite (RC) materials displaying this optical property significantly reduce aesthetic mishaps^5,6^.

Even though a monoshade universal resin composite is considered a suitable option for numerous restorations, it might not be appropriate in every instance, and conventional shade-matching techniques may still be required. It’s crucial to assess the potential limitations of this material in each unique situation, as monoshade universal RCs’ shade-matching capabilities may not be as accurate as those of conventional composite materials^7,8^.

Clinically based studies are constantly introduced to put hands on various factors that cannot be tested in in-vitro research. The interaction of mastication, saliva, change of pH due to various foods and beverages, and temperature changes on dental restorations may alter the protocols dentists follow while applying multiple restorative materials. Since there have been only a few in-vivo studies on single-shade universal RC, more research is needed to evaluate the color matching and clinical efficacy of this type of RC^9,10^.

Thus, the aim of the research was to compare, at baseline and at the three, six, nine, and twelve-month follow-ups, the color matching of the monoshade composite Omnichroma^®^ and its clinical effectiveness, in comparison with the polyshade nanohybrid RC, Tetric‑N‑Ceram^®^, in Class I and II carious lesions. The null supposed hypothesis suggested that there were no significant discrepancies in the clinical effectiveness of monoshade universal RC restorations compared to conventional polyshade nanohybrid RC restorations for class I and II restorations over 12 months. The current study is dedicated to addressing the following question: Does the clinical effectiveness of monoshade universal RC as a posterior restoration surpass that of the conventional polyshade nanohybrid RC, as evaluated using the modified USPHS criteria?

## Methods

### Sample size calculation

The calculation for the sample size was derived from previous studies on the clinical success rate of composite restorations, which indicated a 93% success rate at 12 months^11^. Earlier studies on posterior tooth restorations, utilizing a significance level of 0.05, a power of 80%, and an equivalency margin of 20%, determined a required sample size of 18 restorations. To account for potential dropouts, we targeted a sample of 20 restorations per group, resulting in a total of 40 restorations, given the use of the split-mouth approach.

### Protocol registration

This study underwent registration on www.clinicaltrials.gov, using the protocol’s distinct identification code NCT05500547 on 15/08/2022. This experimental protocol and all investigations comprising human subjects were approved by the Research Ethics Committee, Faculty of Dentistry, Minia University, bearing code number 420 (Ref. no. 27/06/2020) in accordance with the Declaration of Helsinki.

### Study design

The study design adhered to the Consolidated Standards of Reporting Trials (CONSORT) statement^12^. In the present study, a double-blinded clinical trial was conducted, where both patients and examining operators were unaware of the randomization procedure for the inserted restorations. A split-mouth design was implemented, and online software (www.sealedenvelope.com) was employed to determine two parallel groups with a 1:1 allocation ratio. Each patient received two RC restorations where single shade universal RC (Omnichroma) was employed on one side (Group I) and multi-shade RC (Tetric^®^ N-Ceram) was employed on the other side (Group II) randomly.

### Patient selection

The present study included patients who visited the Conservative Department Clinic for dental care. Patient selection continued until reaching 20 patients that fulfilled the inclusion criteria. The obtained written informed consent from each patient clearly outlined the procedure before any procedure was conducted to guarantee their awareness of the study’s purpose. The study included patients whose ages ranged from 20 to 40 years. These patients were suffering from carious lesions, either occlusal or proximal, in collateral posterior teeth. These lesions were clinically identified, and a radiograph was used to evaluate their extent. “As identified by the radiographic examination, only cavities with caries extending no more than half the thickness of the coronal dentin were included. Heavy smokers, patients with deleterious parafunctional habits, bruxism, traumatic occlusion, or those on prescribed antibiotics within six months prior to inclusion in the study, as well as patients taking analgesics that could potentially affect pain perception, those with obvious wear facets on the occlusal surface, those undergoing orthodontic treatment, or those with temporomandibular joint issues, were excluded from the trial. Additionally, pregnant women and medically compromised patients were not considered eligible for the study.

The included patients were required to have satisfactory oral hygiene, with gingival tissues in a healthy condition, no recession or alveolar bone loss around the teeth, and no orofacial pain or sudden pain. Other criteria were applied, such as having opposing natural teeth free of restorations, a normal and complete occlusion, and a good response to electric pulp testing conducted using the Pulp Tester DY 310 with a threshold of 0–39 (Denjoy Dental Co., Ltd., Changsha, China). The pulp vitality testing was applied to the tooth that required a restorative procedure and its contralateral counterpart.

### Randomization and blinding

Using the website www.random.org, a split-mouth design was implemented, and a random list was generated to assign participants to two groups. Sequentially, each patient selected a number from 1 to 20 based on the previously generated random lists. Throughout the study, both the assessing operator and the participating patients remained unaware of their assigned treatment. However, blinding the operator was not possible due to the nature of the shade selection procedure that was required^13^.

### Clinical procedure

Before any procedure was performed, shade selection was completed prior to dehydration, which could adversely affect the color value. Each participant’s teeth were first checked for cleanliness and cleaned if needed. The shade evaluation was conducted during the time frame from 10 a.m. to 1 p.m. The unit lamps were turned off to ensure uniform ambient lighting and make use of natural daylight. To prevent strong contrasts and possible result distortion, a neutrally colored napkin was used to cover the patient’s clothing. Before the session, the participants were asked not to apply any external makeup that could alter the operator’s ability to distinguish different shades. The shade determination was then carried out visually using the VITA Classical A1-D4 shade guide (VITA Zahnfabrik)^14^. Anesthesia was administered to each patient utilizing 1.8 mL of 2% lidocaine hydrochloride combined with phenylephrine (1:2500) (SS White 100, SS White, Petrópolis, Brazil). Rubber dam isolation was achieved with Ivoclar Vivadent’s OptraDam^®^ Plus, and cavity preparation was performed utilizing a #330 high-speed carbide bur (FG, Dentsply Midwest^®^, Germany). Subsequently, the remaining carious tissue was eliminated using tungsten carbide burs (Komet, Brasseler GmbH Co. KG) at a low speed, in addition to sharp excavators (mailfaire 57/58, Switzerland). The cavity preparation was limited to managing decay while preserving sound tooth structure using the selective caries removal technique. Afterward, any irregularities in the enamel walls of the cavity were smoothed utilizing yellow-coded stones (Komet, Germany).

For Class II cavities, an appropriate sectional matrix and wooden wedges were used for matrixing. In the current study, a 36% phosphoric acid gel was applied to selectively etch the enamel for 30 s, followed by rinsing and air drying for 5 s using a gentle, moisture- and oil-free airflow. Afterward, a single drop of adhesive was applied and scrubbed onto the surface for 20 s. The area was then gently air-dried with an oil-free airflow for 3–5 s, following the manufacturer’s instructions. Finally, a 20-second light curing process was performed with direct occlusal contact. Bluephase N wireless light cure with wavelength ranging from 385 to 515 nm and light intensity of 1100 mW/cm2 was used with zero contact to the occlusal surface of the tooth to be restored in the bonding procedure and curing of each resin composite increment. Complete charging of the light-cured device and daily testing before the use of light cure was performed to make sure that the output light intensity is the same each day using the Bluephase meter II (Bluephase Style, Ivoclar-Vivadent, Schaan/Liechtenstein).

**Fig. 1:**
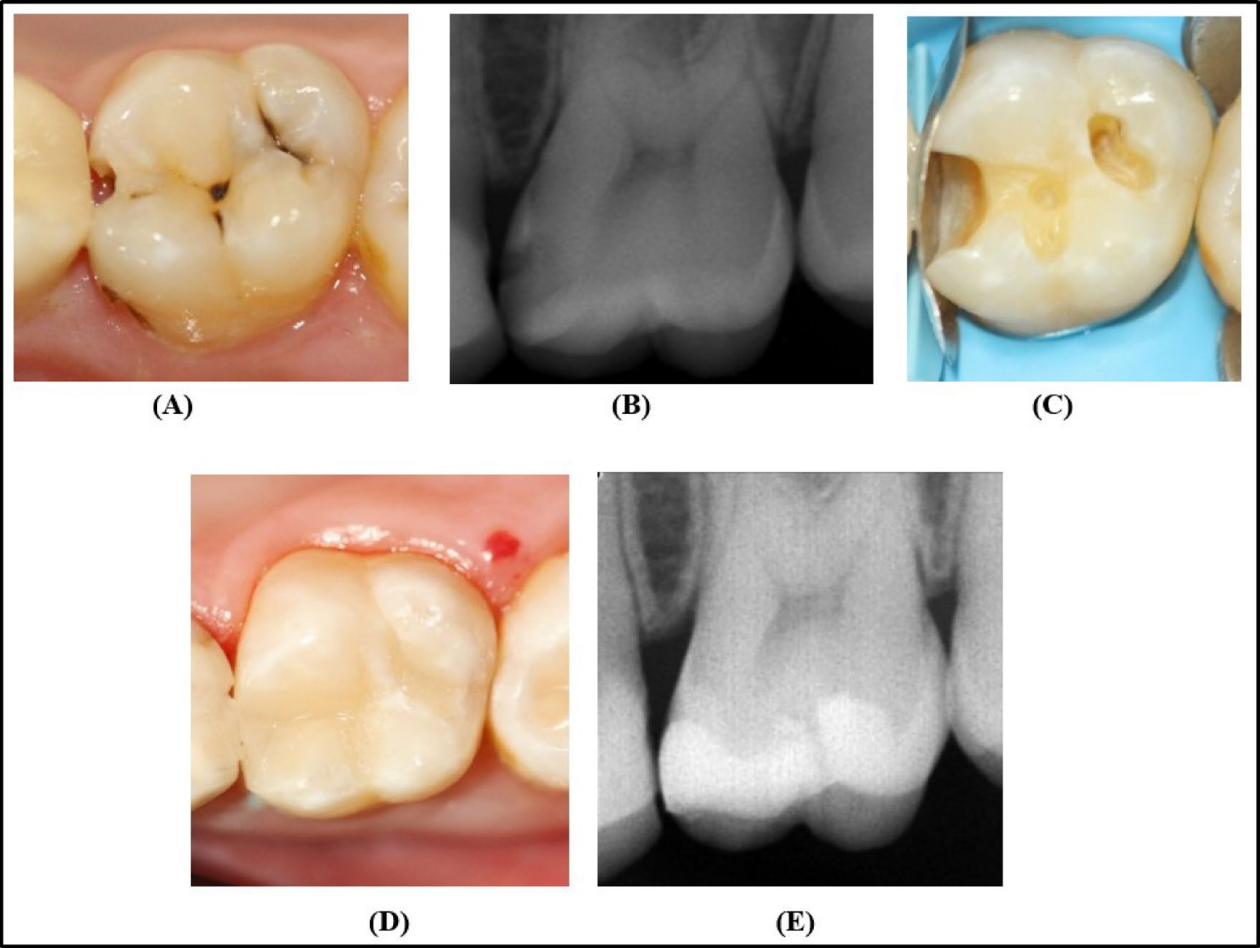
Illustrates the clinical method for cavity preparation and RC restoration; (**A**) Pre-operative photograph, (**B**) Pre-operative radiograph, (**C**) Rubber dam isolation, prepared cavity, wedging and matricing, (**D**) RC restoration after polishing and finishing, (**E**) Post-operative radiograph.

A single-shade universal RC (Omnichroma) and a multi-shade nanohybrid RC (Tetric^®^ N-Ceram) were used (Table 1). RC application was performed strictly following the instructions provided by the respective manufacturers. RC was packed into the prepared cavities using an incremental approach. Each increment was applied in oblique layers, up to 4 layers, in accordance with the cavity size, with a 2 mm maximum thickness per increment. After each increment, 20 s of light curing were applied. During the same appointment, and after the removal of the isolating rubber dam, the restorations underwent meticulous finishing and contouring with fine-grit diamond finishing stones (Komet, Brasseler GmbH Co. KG) at low speed and with ample water. Additionally, articulating paper (Bausch, Nashua, NH, USA) was employed to check for premature contacts in centric and eccentric occlusion, sequentially using 100 *µM* and 40 *µM*^15^. The restorations were promptly polished utilizing EVE DIACOMP Plus OccluFlex-impregnated rubber cups and impregnated brushes (Optishine, Kerr Switzerland) with a water coolant. To achieve a smooth surface, Sof-Lex discs (3 M ESPE, Germany) were used in the prescribed sequence of coarse, medium, fine, and superfine, accompanied by a water coolant, to finish the buccal and lingual embrasures in Class II cavities. The first (the black disc) was used only when there was bulky buccal or lingual restorative material, to reduce the time required for finishing and polishing Fig. 1.

**Table 1:** Illustrates the materials employed in the present study.

Material	Composition	Manufacturer	Lot #
Omnichroma	Matrix: TEGDMA^(1)^, UDMA^(2)^, Dibutyl hydroxyl toluene and UV^(3)^ absorber, Mequinol. Filler system: SiO_2_, ZrO_2_ (68 vol.-%; 79 wt%; 0.2–0.4 μm)	Tokuyama Dental, Tokio, Japan www.tokuyama-dental.com	002E60
Palfique bond Adhesive system	Phosphoric acid monomer, Bis-GMA, TEGDMA ^(6)^, HEMA ^(7)^, camphorquinone, alcohol, and purified water.		176EY0
Tetric^®^ N-Ceram Nano-hybrid incremental composite	Matrix: BisGMA^(4)^ and DMA^(5)^ (19–20 wt%). Filler system: mixed oxide, barium glass, ytterbium trifluoride, and Copolymers (80–81 wt%). Supplementary components such as catalysts, stabilizers, additives, and pigments are comprised in dental materials, typically comprising less than 1% by weight The dental material includes around 55–57% by volume of inorganic fillers, which have particle sizes with ranges from 40 nm to 3000 nm.	IvoclarVivadent, Schaan, Liechtenstein www.ivoclar.com	Z0158P
AdheSE adhesive: Tetric N-bond Universal	Bis-GMA 25–50%, ethanol 10-<25%, phosphonic acid acrylate 10-<25%, urethane dimethacrylate 3-<10%, diphenyl (2,4,6- trimethyl benzoyl) phosphine oxide 1-<2.5%		Z00B7M

^(1)^ Tri-ethylene glycol methacrylate ^(2)^ Urethane dimethacrylate ^(3)^ Ultra-violet absorber ^(4)^Bisphenol-A glycidyl ether dimethacrylate ^(5)^ Dimethacrylate ^(6)^Triethylene glycol dimethacrylate ^(7)^ 2-Hydroxyethyl.

### Clinical evaluation

All restorations were handled by one experienced operator from the research team. However, for the clinical assessment of the restorations performed, two independent and experienced dentists who were not part of the restorative procedure evaluated the restorations at 0, 1, 3, 6, 9, and 12 months utilizing the modified USPHS criteria. During the follow-up period, restorations were assessed for color match and color stability, anatomic form, retention, surface texture, marginal discoloration, marginal adaptation, contact surface, secondary caries, and patient acceptability. They used a mirror, a probe, and a magnifying loupe with a magnification power of 3.5x (***Ziess***,*** Germany)***. To ensure optimal visibility, the magnifying loupe was equipped with a powerful light source ***(Ziess Eyemag Light II LED illumination system***,*** Ziess***,*** Germany)***.

After completing the final finishing and polishing, a photographic evaluation was performed to assess the achieved shade matching in both groups. A DSLR camera (Canon 700D) with a Sigma 105 mm f/2.8 EX DG OS HSM macro lens, a close-up Speedlight ring flash (Yongnuo, Shenzhen Yongnuo Photographic Equipment Co., Ltd., Shenzhen, China), contractors, retractors (cheek and lip), and mirrors were used. At each follow-up appointment, clinical intraoral photographs and radiographs were captured, and the USPHS parameters were recorded utilizing a standardized case report for each patient during the evaluation procedures. Additionally, each restoration was evaluated using a compressed air stream directed toward the restoration (2–3 cm away) to assess postoperative sensitivity, with proper isolation of neighboring teeth. This process lasted for three seconds, followed by gently running a probe across the restored tooth surface^16^. To determine their clinical acceptability, a scoring system was employed, with Alfa and Bravo indicating exceptional and clinically sufficient findings, Charlie for clinically unacceptable findings that require replacement, and Delta representing clinical failures such as retention loss, significant marginal weaknesses, or discoloration necessitating immediate replacement or repair **(**Table 2).

**Table 2:** Modified USPSH criteria.

Category	Scores	Criteria
Color match (CM)	Alpha Bravo Charlie	Matches tooth. Acceptable mismatch. Unacceptable mismatch.
Retention (R)	Alpha Charlie	No loss of restorative material. Any loss of restorative material.
Anatomic form (AF)	Alpha Bravo Charlie	Continuous. Slight discontinuity, clinically acceptable. Discontinuous, failure.
Surface texture	Alpha Bravo Charlie	Smooth surface comparable to surrounding enamel. Rougher surface compared to the surrounding enamel. Very tough surface.
Marginal adaptation (MA)	Alpha Bravo Charlie	Closely adapted, no detectable margin. Detectable margin clinically acceptable. Marginal crevice clinical failure.
Marginal discoloration (MD)	Alpha Bravo Charlie	No discoloration. Discoloration without axial penetration. Discoloration with axial penetration.
Secondary caries (SC)	Alpha Charlie	No caries present. Caries present.
Post-operative sensitivity	Alpha Bravo Charlie	Not present, Sensitive but diminished in intensity. Constant sensitivity, not diminished in intensity.
Contact surfaces	Alpha Bravo Charlie	Normal Light None
Patient complaints	Alpha Bravo Charlie	Satisfied A minor criticism of aesthetics Desire for improvement
Patient Satisfaction	Alpha Charlie	Entirely satisfied Completely dissatisfied

### Statistical analysis

The percentages and frequencies were employed to present the qualitative data. A comparison of the clinical evaluation scores among the two groups was conducted employing the Wilcoxon signed-rank test. The temporal changes within every group were investigated utilizing Friedman’s test. The numerical data underwent assessment for normality by investigating the data distribution and conducting normality tests, including the Shapiro-Wilk and Kolmogorov-Smirnov tests. All color change data exhibited a non-normal (non-parametric) distribution. The data were presented using measures such as mean, standard deviation (SD), median, and range values. The Wilcoxon signed-rank test was used to compare the two groups in terms of non-parametric data. The statistical analysis was conducted utilizing IBM SPSS Statistics for Windows, Version 23.0, developed by IBM Corp in Armonk, NY. A significance level of *P* ≤ 0.05 was established, indicating the threshold for statistical significance.

## Result

**Fig. 2:**
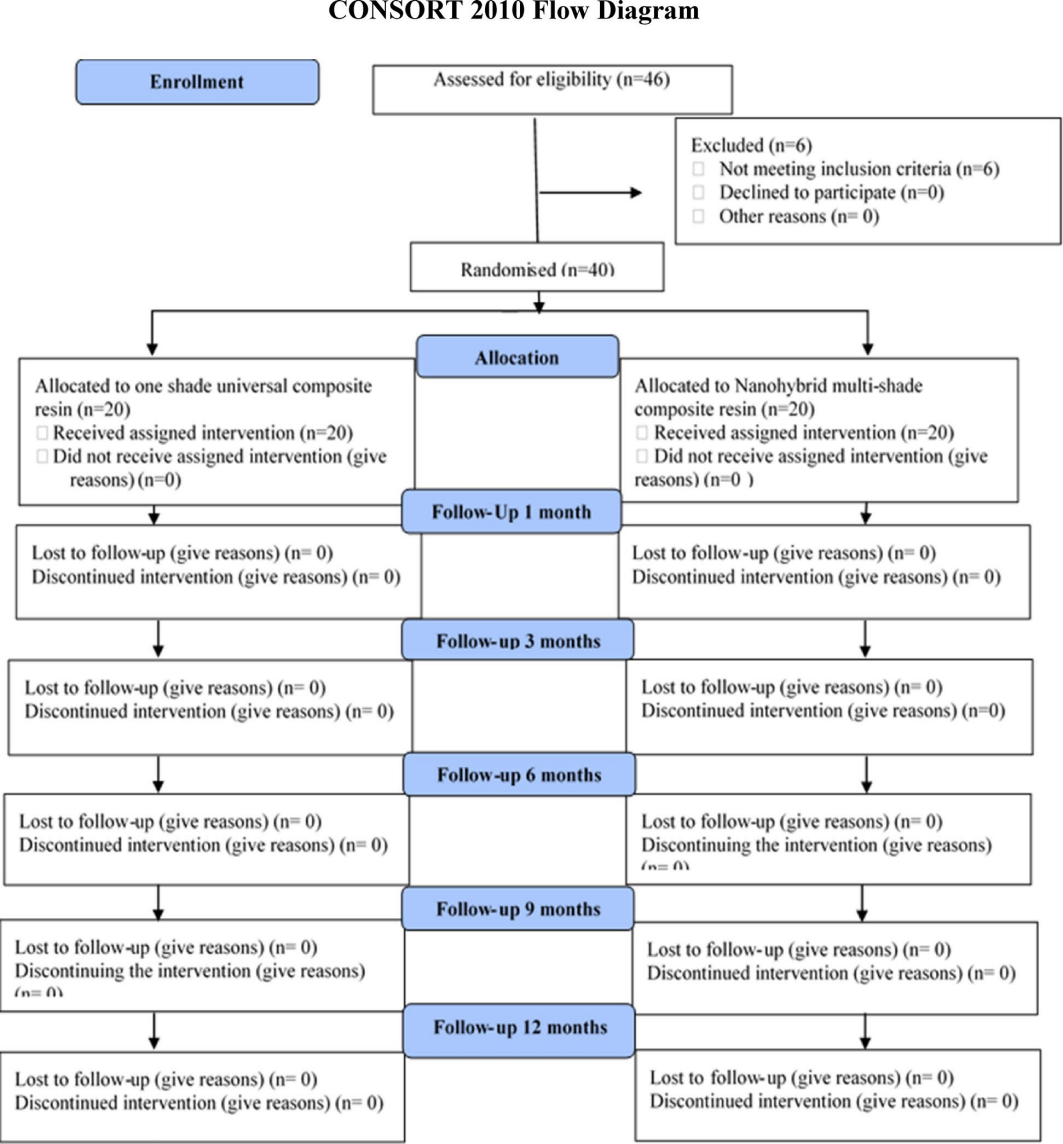
Consort Flowchart: allocation, enrollment & follow-up of patients in the study.

**Fig. 3:**
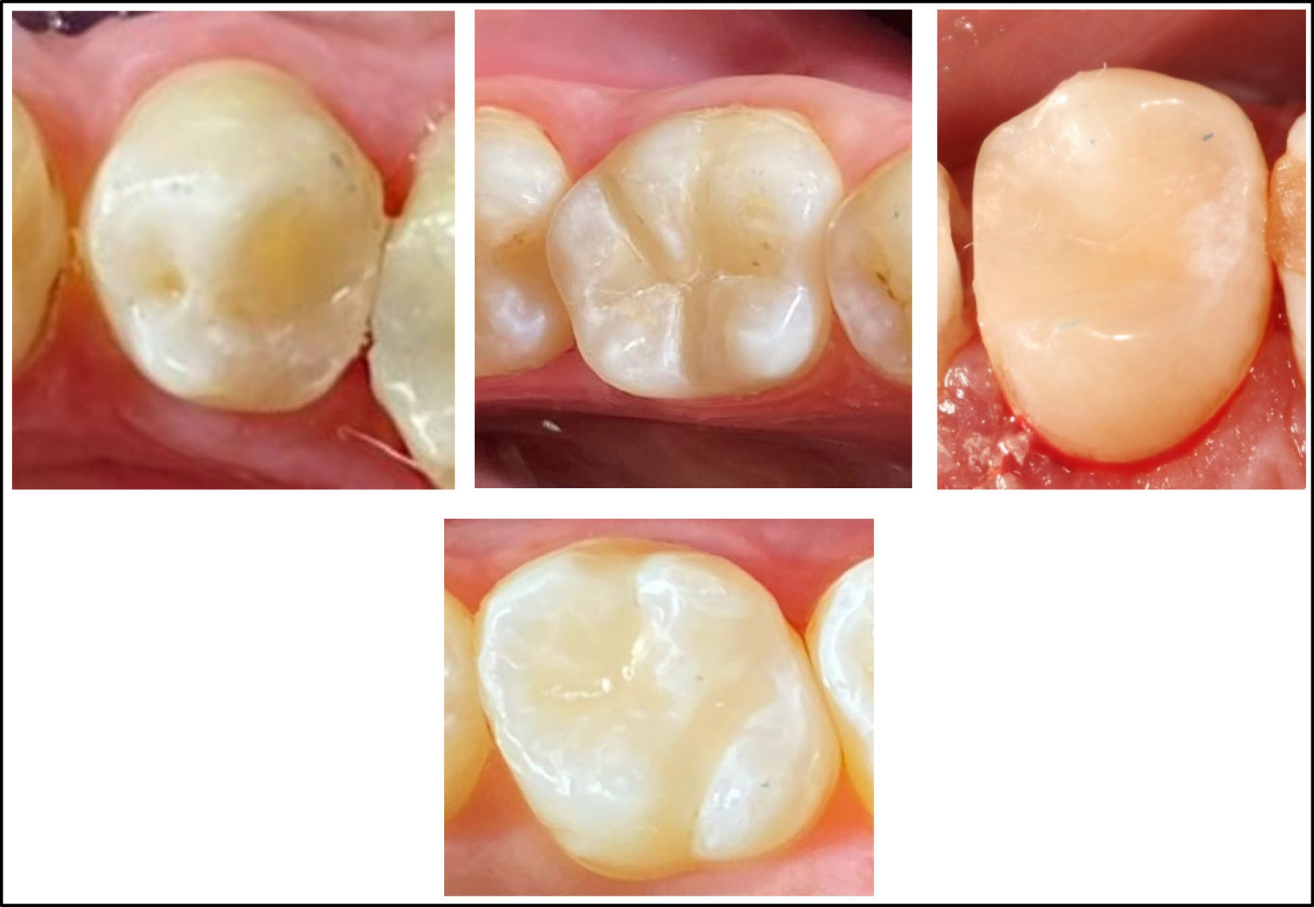
Showing the immediate clinical pictures of teeth restored with Omnichroma.

**Fig. 4:**
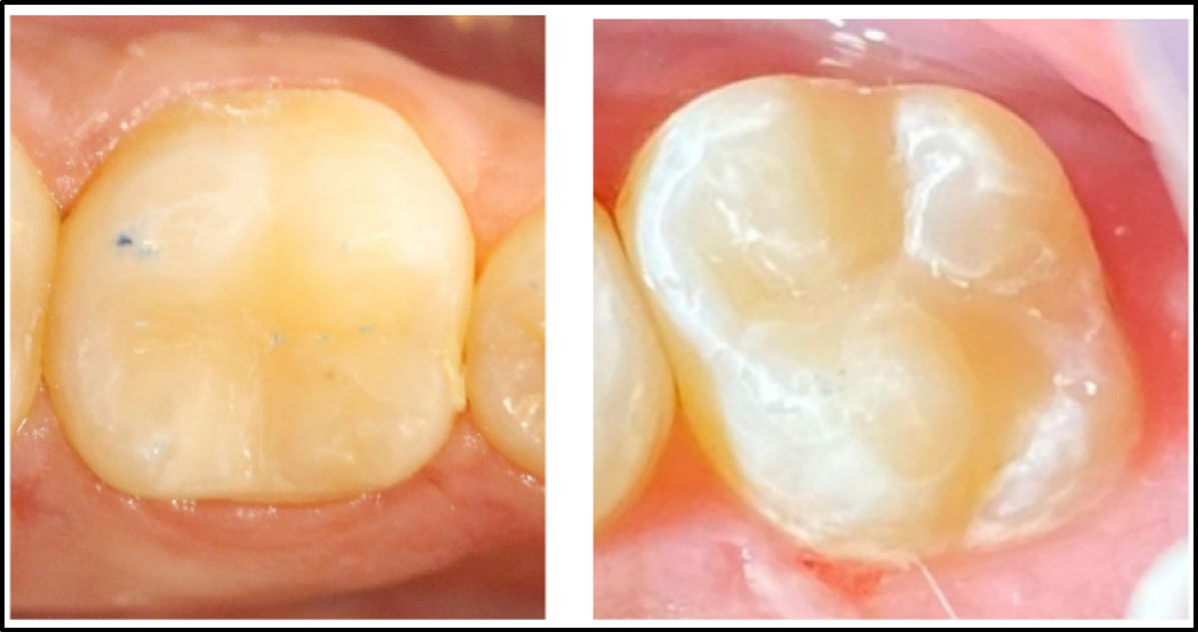
Showing the immediate clinical pictures of teeth restored with Tetric N.

**Fig. 5:**
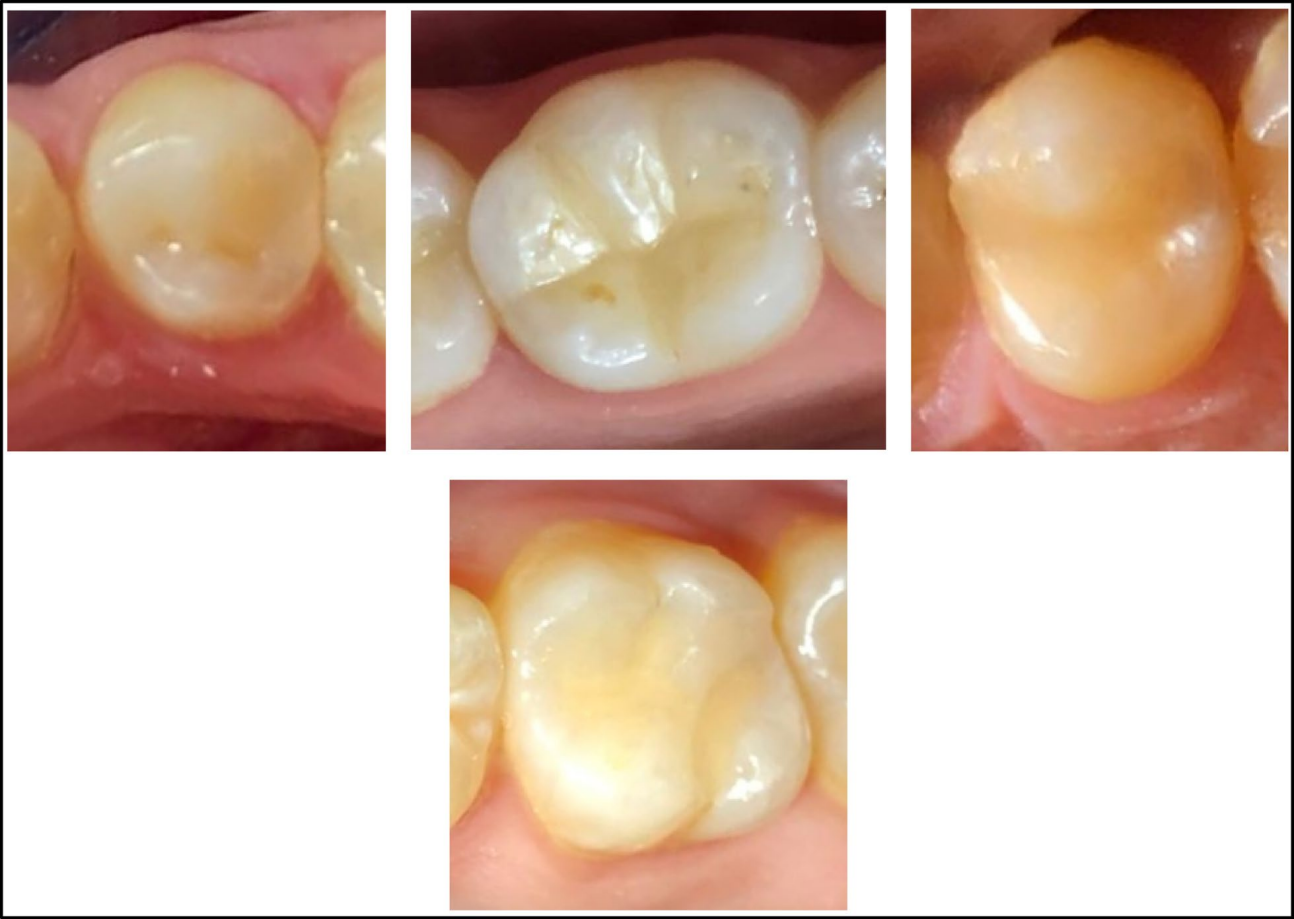
Omnichroma after 12 months of follow-up.

**Fig. 6:**
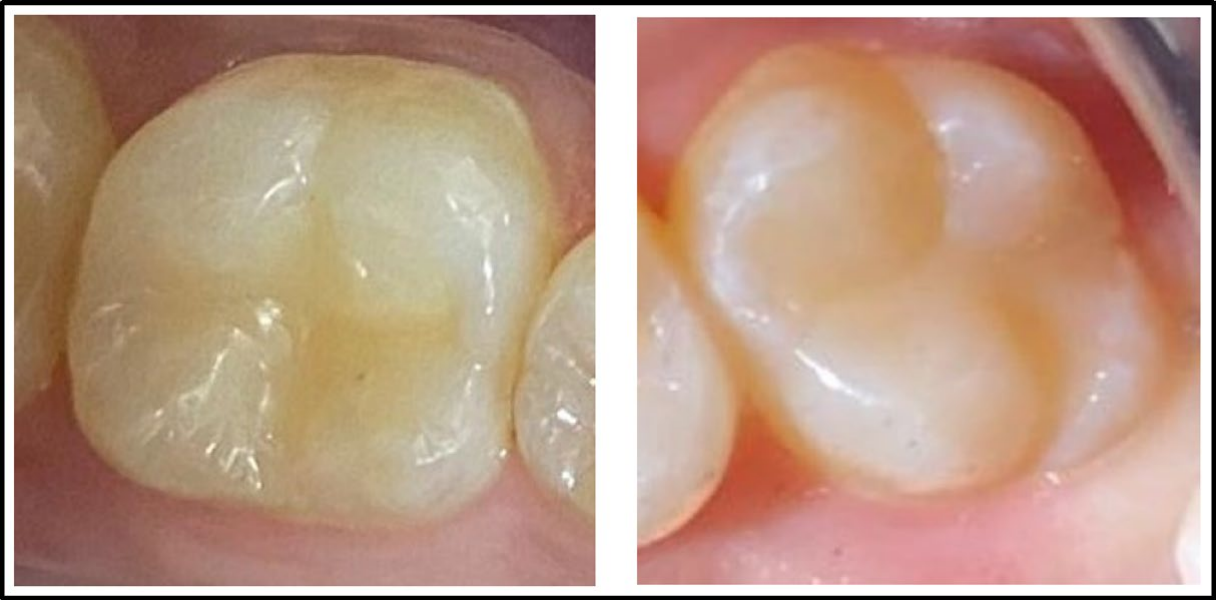
Tetric N After 12 months of follow-up.

Prior to performing the current study, a pre-calibration process was conducted with 10 patients who were not part of the study, ensuring a reliability of 90%. The examiners demonstrated a noteworthy level of agreement, ranging from substantial to nearly ideal (Table 3). Of the twenty patients treated, 13 were males (65%), while 7 were females (35%). Demographic analysis for the distribution of teeth and the classification of cases displayed no statistically significant difference among the two groups (P = 0.750, 0.655), respectively (Table 4). A 100% recall rate was achieved during the 12-month recall period, Fig. 2. Tables 5 and 6 present the clinical evaluation findings conducted during the 12-month monitoring period according to the modified USPHS. 100% of the restorations were found to lie among the Alpha and Bravo scores, with no statistically significant difference among the two groups, except at nine and 12 months for color stability and color match. In these cases, 15 restorations from the Omnichroma group received Bravo scores (75%), which showed a statistically significant difference (P = 0.025) in comparison to the Tetric N group. No significant differences were identified between baseline and 12 months (P > 0.05), given that all restorations in both groups achieved (Alpha) scores for retention, recurrent caries, marginal discoloration, and marginal adaptation. Meanwhile, there were insignificant differences in the surface texture and anatomic form between the tested groups at baseline, one month, three month, and six months. At both the nine and 12-month follow-up periods, 5% of patients showed (Bravo) scores in the Omnichroma group, with no statistically significant differences marked between both groups (P = 0.317). Concerning postoperative sensitivity, after three, six, nine, and 12 months, 5 − 10% of patients showed (Bravo) scores in both groups with no statistically significant differences (P = 0.317, 0.157) observed among both groups Figure (3, 4, 5, 6).

**Table 3:** Reliability test.

	Interobserver reliability			Intra-observer reliability		
	Cronbach alpha	ICC	*P* value	Cronbach alpha	ICC	*P* value
**Color match and color stability scores**	0.947	0.899	< 0.001*	0.975	0.952	< 0.001*
**Retention scores**	0.894	0.808	< 0.001*	0.787	0.749	< 0.001*
**Anatomic form scores**	0.963	0.823	< 0.001*	0.895	0.752	< 0.001*
**Surface texture scores**	0.932	0.898	< 0.001*	0.867	0.852	< 0.001*
**Marginal adaptation scores**	0.935	0.872	< 0.001*	0.985	0.723	< 0.001*
**Marginal discoloration scores**	0.896	0.842	< 0.001*	0.911	0.841	< 0.001*
**Contact surface scores**	0.963	0.872	< 0.001*	0.901	0.836	< 0.001*
**Secondary caries scores**	0.867	0.825	< 0.001*	0.865	0.748	< 0.001*
**Post-operative sensitivity scores**	0.936	0.752	< 0.001*	0.864	0.763	< 0.001*
**Patient complaint**	0.987	0.821	< 0.001*	0.964	0.872	< 0.001*
**Patient satisfaction**	0.912	0.870	< 0.001*	0.856	0.756	< 0.001*

Based on the reliability test results, both inter-observer and intra-observer reliability demonstrate excellent consistency across all evaluation parameters. Inter-observer reliability shows particularly strong agreement, with Cronbach’s alpha values ranging from 0.867 to 0.987, indicating exceptional internal consistency among different raters. The corresponding ICC values (0.752–0.899) further confirm substantial to almost perfect agreement between observers. Similarly, intra-observer reliability exhibits high consistency, with Cronbach’s alpha values ranging from 0.787 to 0.985, suggesting strong repeatability within individual raters’ assessments. ICC values for intra-observer reliability (0.723–0.952) indicate good to excellent consistency in repeated measurements by the same observer. All reliability measures achieved statistical significance (*p* < 0.001), providing robust evidence that the assessment methodology is highly reliable and reproducible. Notably, parameters such as “Color match and color stability” and “Patient complaint” show the strongest consistency across both reliability measures, while “Retention scores” and “Post-operative sensitivity” demonstrate relatively lower, though still significantly strong, reliability coefficients. These comprehensive reliability results validate the assessment tool as a dependable instrument for evaluating dental restorations across multiple clinical parameters.

**Table 4:** Frequencies (n), percentages, and the findings of the Wilcoxon signed-rank test were analyzed to compare demographic data between the two groups.

	Omnichroma (*n* = 20)		Tetric *N*		*P*-value
	*N*	%	*n*	%	
**Tooth**					
Mandibular premolars	1	5	1	5	
Mandibular molars	11	55	14	70	
Maxillary premolars	3	15	3	5	
Maxillary molars	5	25	4	20	
**Class**					
Class I	16	80	17	85	0.655
Class II	4	20	3	15	

*: Significant at *P* ≤ 0.05.

## Discussion

The main objective of esthetic dentistry is to attain visual characteristics that closely mimic the appearance of natural enamel and dentin, ensuring that dental restorations blend seamlessly with their surroundings and remain virtually undetectable. Furthermore, it replicates not only just the visual attributes but also the physical attributes of enamel and dentin, ensuring their functional suitability^2^. The current study aimed to compare the clinical effectiveness and color matching of monoshade universal RC versus polyshade RC in the treatment of class I and II carious lesions over a one-year follow-up period.

**Table 5:** The descriptive statistics and outcomes of the Wilcoxon signed-rank test were utilized to compare retention, anatomic form, postoperative hypersensitivity scores, surface texture, secondary caries, and marginal adaptation in both groups.

Recall time (months)				0	1	3	6	9	12		0	1	3	6	9	12		0	1	3	6		9		12		
Restorative system				**Alpha**							**Bravo**							**Charlie**									
Color match and color stability scores																											
Omnichroma (*n* = 20)	n			20	20	19	18	15	15		0	0	1	2	5	5		0	0	0	0		0		0		
	%			100	100	95	90	75	75		0	0	5	10	25	25		0	0	0	0		0		0		
Tetric N (*n* = 20)	n			20	20	20	20	20	20		0	0	0	0	0	0		0	0	0	0		0		0		
	%			100	100	100	100	100	100		0	0	0	0	0	0		0	0	0	0		0		0		
*P-value*				1	1	0.317	0.157	0.025*	0.025*																		
Retention scores																											
Omnichroma (*n* = 20)	n			20	20	20	20	20	20		0	0	0	0	0	0		0	0	0	0		0		0		
	%			100	100	100	100	100	100		0	0	0	0	0	0		0	0	0	0		0		0		
Tetric N (*n* = 20)	n			20	20	20	20	20	20		0	0	0	0	0	0		0	0	0	0		0		0		
	%			100	100	100	100	100	100		0	0	0	0	0	0		0	0	0	0		0		0		
*P-value*			***Not computed†***																								
Anatomic form scores																											
Omnichroma (*n* = 20)	n			20	20	20	20	19	19		0	0	0	0	1	1		0	0	0	0		0		0		
	%			100	100	100	100	95	95		0	0	0	0	5	5		0	0	0	0		0		0		
Tetric N (*n* = 20)	n			20	20	20	20	20	20		0	0	0	0	0	0		0	0	0	0		0		0		
	%			100	100	100	100	100	100		0	0	0	0	0	0		0	0	0	0		0		0		
*P-value*				1	1	1	1	0.317	0.317																		
Surface texture scores																											
Omnichroma (*n* = 20)	n			20	20	20	20	19	19		0	0	0	0	1	1		0	0	0	0		0		0		
	%			100	100	100	100	95	95		0	0	0	0	5	5		0	0	0	0		0		0		
Tetric N (*n* = 20)	n			20	20	20	20	20	20		0	0	0	0	0	0		0	0	0	0		0		0		
	%			100	100	100	100	100	100		0	0	0	0	0	0		0	0	0	0		0		0		
*P-value*				1	1	1	1	0.317	0.317																		
Marginal adaptation scores																											
Omnichroma (*n* = 20)	n			20	20	20	20	20	20		0	0	0	0	0	0		0	0	0	0		0		0		
	%			100	100	100	100	100	100		0	0	0	0	0	0		0	0	0	0		0		0		
Tetric N (*n* = 20)	n			20	20	20	20	20	20		0	0	0	0	0	0		0	0	0	0		0		0		
	%			100	100	100	100	100	100		0	0	0	0	0	0		0	0	0	0		0		0		
*P-value*			***Not computed†***																								
Marginal discoloration scores																											
Omnichroma (*n* = 20)	n			20	20	20	20	20	20		0	0	0	0	0	0		0	0	0		0		0	0		
	%			100	100	100	100	100	100		0	0	0	0	0	0		0	0	0		0		0	0		
Tetric N (*n* = 20)	n			20	20	20	20	20	20		0	0	0	0	0	0		0	0	0		0		0	0		
	%			100	100	100	100	100	100		0	0	0	0	0	0		0	0	0		0		0	0		
*P-value*			***Not computed†***																								
Contact surface scores																											
Omnichroma (*n* = 20)	n			20	20	20	20	20	20		0	0	0	0	0	0		0	0	0	0		0		0		
	%			100	100	100	100	100	100		0	0	0	0	0	0		0	0	0	0		0		0		
Tetric N (*n* = 20)	n			20	20	20	20	20	20		0	0	0	0	0	0		0	0	0	0		0		0		
	%			100	100	100	100	100	100		0	0	0	0	0	0		0	0	0	0		0		0		
*P-value*			***Not computed†***																								
Secondary caries scores																											
Omnichroma (*n* = 20)	n			20	20	20	20	20	20		0	0	0	0	0	0		0	0	0	0		0		0		
	%			100	100	100	100	100	100		0	0	0	0	0	0		0	0	0	0		0		0		
Tetric N (*n* = 20)	n			20	20	20	20	20	20		0	0	0	0	0	0		0	0	0	0		0		0		
	%			100	100	100	100	100	100		0	0	0	0	0	0		0	0	0	0		0		0		
*P-value*		***Not computed†***																									
Post-operative sensitivity scores																											
Omnichroma (*n* = 20)	n			20	20	19	18	19	19		0	0	1	2	1	1		0	0	0	0		0		0		
	%			100	100	95	90	95	95		0	0	5	10	5	5		0	0	0	0		0		0		
Tetric N (*n* = 20)	n			20	20	19	19	20	20		0	0	1	1	0	0		0	0	0	0		0		0		
	%			100	100	95	95	100	100		0	0	5	5	0	0		0	0	0	0		0		0		
*P-value*				1	1	0.317	0.157	0.317	0.317																		
Patient complaint																											
Omnichroma (*n* = 20)	n			20	19	18	18	19	20		0	1	2	2	1	0		0	0	0	0		0		0		
	%			100	95	90	90	95	100		0	5	10	10	5	0		0	0	0	0		0		0		
Tetric N (*n* = 20)	n			20	20	20	20	20	20		0	0	0	0	0	0		0	0	0	0		0		0		
	%			100	100	100	100	100	100		0	0	0	0	0	0		0	0	0	0		0		0		
*P-value*				1	0.317	0.157	0.157	0.317	1																		
Patient satisfaction																											
Omnichroma (*n* = 20)	n			20	20	20	20	20	20		0	0	0	0	0	0		0	0	0	0		0		0		
	%			100	100	100	100	100	100		0	0	0	0	0	0		0	0	0	0		0		0		
Tetric N (*n* = 20)	n			20	20	20	20	20	20		0	0	0	0	0	0		0	0	0	0		0		0		
	%			100	100	100	100	100	100		0	0	0	0	0	0		0	0	0	0		0		0		
*P-value*		***Not computed†***																									

*: Significant at *P* ≤ 0.05, ^†^: Not computed because the variable is constant.

**Table 6:** Descriptive statistics and friedman’s test were employed to analyze the post-operative sensitivity scores, anatomic form, and surface texture at different intervals of follow-up for every group.

Material	Parameter		Time (months)	Scores			*P-value*
				**Alpha**,** n (%)**	**Bravo**,** n (%)**	**Charlie**,** n (%)**	
Omnichroma	**Color stability**		**1**	**20 (100)**	**0 (0)**	**0 (0)**	0.004*
			**3**	**19 (95)**	**1 (5)**	**0 (0)**	
			**6**	**18 (90)**	**2 (10)**	**0 (0)**	
			**9**	**15 (75)**	**5 (25)**	**0 (0)**	
			**12**	**15 (75)**	**5 (25)**	**0 (0)**	
Tetric N			**1**	**20 (100)**	**0 (0)**	**0 (0)**	**Not computed** ^**†**^
			**3**	**20 (100)**	**0 (0)**	**0 (0)**	
			**6**	**20 (100)**	**0 (0)**	**0 (0)**	
			**9**	**20 (100)**	**0 (0)**	**0 (0)**	
			**12**	**20 (100)**	**0 (0)**	**0 (0)**	
Omnichroma	**Surface texture**		**1**	**20 (100)**	**0 (0)**	**0 (0)**	**0.406**
			**3**	**20 (100)**	**0 (0)**	**0 (0)**	
			**6**	**20 (100)**	**0 (0)**	**0 (0)**	
			**9**	**19 (95)**	**1 (5)**	**0 (0)**	
			**12**	**19 (95)**	**1 (5)**	**0 (0)**	
Tetric N			**1**	**20 (100)**	**0 (0)**	**0 (0)**	**Not computed** ^**†**^
			**3**	**20 (100)**	**0 (0)**	**0 (0)**	
			**6**	**20 (100)**	**0 (0)**	**0 (0)**	
			**9**	**20 (100)**	**0 (0)**	**0 (0)**	
			**12**	**20 (100)**	**0 (0)**	**0 (0)**	
Omnichroma	**Anatomic form**		**1**	**20 (100)**	**0 (0)**	**0 (0)**	**0.406**
			**3**	**20 (100)**	**0 (0)**	**0 (0)**	
			**6**	**20 (100)**	**0 (0)**	**0 (0)**	
			**9**	**19 (95)**	**1 (5)**	**0 (0)**	
			**12**	**19 (95)**	**1 (5)**	**0 (0)**	
Tetric N			**1**	**20 (100)**	**0 (0)**	**0 (0)**	**Not computed** ^**†**^
			**3**	**20 (100)**	**0 (0)**	**0 (0)**	
			**6**	**20 (100)**	**0 (0)**	**0 (0)**	
			**9**	**20 (100)**	**0 (0)**	**0 (0)**	
			**12**	**20 (100)**	**0 (0)**	**0 (0)**	
Omnichroma	**Post-operative sensitivity**		**1**	**20 (100)**	**0 (0)**	**0 (0)**	**0.504**
			**3**	**19 (95)**	**1 (5)**	**0 (0)**	
			**6**	**18 (90)**	**2 (10)**	**0 (0)**	
			**9**	**19 (95)**	**1 (5)**	**0 (0)**	
			**12**	**19 (95)**	**1 (5)**	**0 (0)**	
Tetric N			**1**	**20 (100)**	**0 (0)**	**0 (0)**	**0.406**
			**3**	**19 (95)**	**1 (5)**	**0 (0)**	
			**6**	**19 (95)**	**1 (5)**	**0 (0)**	
			**9**	**20 (100)**	**0 (0)**	**0 (0)**	
			**12**	**20 (100)**	**0 (0)**	**0 (0)**	
Omnichroma	**Patient complaint**	**1**		**20 (100)**	**0 (0)**	**0 (0)**	**0.504**
		**3**		**19 (95)**	**1 (5)**	**0 (0)**	
		**6**		**18 (90)**	**2 (10)**	**0 (0)**	
		**9**		**19 (95)**	**1 (5)**	**0 (0)**	
		**12**		**20 (100)**	**0 (0)**	**0 (0)**	
Tetric N		**1**		**20 (100)**	**0 (0)**	**0 (0)**	**Not computed** ^**†**^
		**3**		**20 (100)**	**0 (0)**	**0 (0)**	
		**6**		**20 (100)**	**0 (0)**	**0 (0)**	
		**9**		**20 (100)**	**0 (0)**	**0 (0)**	
		**12**		**20 (100)**	**0 (0)**	**0 (0)**	

*: Significant at *P* ≤ 0.05.

Studying the clinical performance of monoshade universal RC with structural coloring in posterior teeth provides valuable knowledge for clinicians, enabling them to advocate for dental materials that mimic and harmonize with natural tooth shades. This simplifies shade selection and reproduction, ensures patient satisfaction, and enhances aesthetic dental procedures. Limited research has been conducted on the clinical effectiveness of Omnichroma, and currently, there is insufficient in vivo evidence regarding its efficacy. Consequently, this clinical trial was conducted to assess and contrast the clinical efficacy of Omnichroma with Tetric-N-Ceram in permanent posterior teeth using USPHS criteria. These criteria allow for valid comparisons among studies with different observation periods, and modified versions of these criteria are still widely utilized to assess different aspects of restorations^17,18^.

The hypothesis was accepted as the follow-up data collected after one year indicated that both materials performed similarly. The majority of scores for the restorative materials used in this study fell within the acceptable range of Alpha to Bravo, indicating satisfactory outcomes.

The current findings concerning the color stability and color match revealed no significant difference between the two RC materials tested throughout the whole recall period. This outcome can be ascribed to the smart chromatic technology of the resin composites, which reflects the colors encompassing the spectrum from red to yellow, which occur in all teeth. This innovative technology relies on the utilization of fillers (uniform supra-nano round and spherical fillers composed of silica and zirconia particles), which are hypothesized to generate structural color spanning from red to yellow when light interacts with them. The surrounding tooth color is combined with these colors to create an unparalleled color matching^19^.

Omnichroma has a remarkable ability to optically mimic the surrounding structure without any pigments integrated into the material. This optical behavior is achieved through the nanofillers’ ability to selectively interact with specific light wave frequencies, resulting in the precise reflection of a distinct wavelength within the natural tooth color spectrum^20^. To achieve this optical ability, the RC filler must contain only uniform, spherical particles of precise dimension^21^.

These results were confirmed by Ebaya et al., who stated that monoshade universal resin composites demonstrate acceptable surface smoothness and effectively match the color of various tooth shades^19^. Consequently, the composite, after curing, integrates with the adjacent tooth structure. These results are in accordance with Sensi et al., who proved that Omnichroma exhibited minimal discoloration upon exposure to simulated aging^22^.

Following the initial 9-month assessment duration, some structurally colored universal resin composite restorations in a monoshade exhibited a shift to the Bravo score regarding color stability but remained clinically acceptable. This finding aligns with the results reported by de Abreu et al. and Iyer et al.^23,24^, who specified that the monoshade RC exhibits inferior color matching compared to polyshade RC, which may limit its clinical applicability in cases with superior aesthetic demands. Moreover, Ebaya et al. and Forabosco et al.^19,25^ verified that the aging technique had an adverse impact on the surface characteristics color stability of universal RCs, suggesting that water absorption affects the mechanical characteristics of composites, resulting in hydrolytic decomposition. In addition, it can potentially lead to micro-fractures at the filler/resin matrix interface and cause superficial stress because of significant temperature gradient fluctuations. These factors can ultimately impact the surface roughness and increase the composite’s susceptibility to stain absorption.

The patient’s primary goal in assessing the quality of aesthetic treatment is to achieve accurate color matching among the dental restoration and the natural dental structure. The recent development of restorative systems incorporating multiple shades and varying translucency levels—high, medium, and low-value enamel shade RCs—stems from the pursuit of more realistic and natural-looking restorations^26^. Omnichroma^®^, a material capable of achieving a wide range of color matching, was selected for its smart chromatic technology, which controls its optical properties. Its arrangement of filler particles corresponds to the wavelengths of visible light, which helps limit color distortion and minimize shade variation over time by reducing photochemical deterioration^27,28^. The mechanical and physical characteristics of a nanohybrid RC called Tetric N-Ceram are influenced by the presence of nanofillers, including nanomers and nanoclusters, which act as nanomodifiers^29,30^.

The selection of additional criteria for assessing the clinical efficacy of the RCs, such as retention, postoperative sensitivity, secondary caries, contact surface, marginal discoloration, and marginal adaptation, was based on their association with polymerization shrinkage^31^.

Omnichroma and TetricNCeram performed similarly over 12 months with no statistically significant difference for the assessed clinical criteria except for post-operative sensitivity. After three, six, nine, and twelve months, 5 − 10%, for groups I and II, respectively, patients showed (Bravo) scores in both groups with no statistically significant difference observed between both groups.

In terms of retention, the results of the current study were confirmed by the research carried out by Zulekha et al.^1^, who demonstrated that the similar amount of filler content may explain the comparable results for both types of RCs, which reduces polymerization shrinkage, using the same clinical technique, isolation protocol, bonding agent, and exposure to the similar oral environment.

Based on a meta-analysis, it was found that conventional RC restorations exhibited superior marginal adaptation compared to RC restorations with new and modified monomers after a 12-month follow-up. However, for more extended follow-up periods, no considerable variations were noted among the two types of restorations^24^.

Conflicting outcomes were presented by Bajabaa et al.^32^, where Omnichroma exhibited greater microleakage than Tetric‑N‑Ceram. This could be attributed to the inclusion of TEGDMA in the Omnichroma resin matrix, which has a lower molecular weight than Bis‑GMA and UDMA in Tetric‑N‑Ceram. Consequently, this significantly exacerbates microleakage and polymerization shrinkage. Microleakage can lead to stain penetration, water sorption, and eventually discoloration.

The patient complaints in the current study showed that 5–10% of their complaints were related to postoperative sensitivity (group I and II, respectively), which decreased over the follow-up period. Kruly et al. and Rajnekar et al.^31,33^ reported that postoperative sensitivity can arise due to the resin composites’ polymerization shrinkage stress. Excessive stress during polymerization poses a challenge to the bonded interface, as it can lead to the formation of cracks in the dentin on the pulpal floor. Despite implementing alterations in tooth surface treatments, making changes to polymerization methods, and adopting incremental placement techniques for composite restorations, postoperative sensitivity remains a challenging and unresolved problem encountered by patients.

The polishability and polish retention may result from the specific alignment of the filler particles. Kim et al.^34,35^ found that, after finishing, Omnichroma exhibited a notably smoother surface than Tetric N-Ceram. Adhering to the guidelines provided by the manufacturer, the exceptional polishability of Omnichroma can be attributed to the use of uniform spherical fillers at the nano scale, along with consistent spacing between the filler particles. In contrast, Khairy et al.^36^ conducted a study that yielded comparable results concerning the surface roughness of the Nano-fill composite (Omnichroma) in comparison to the Nano-hybrid composite. The findings suggested that the roughness of Omnichroma tends to be higher than that observed in Nano-hybrid composites. Modifying the filler’s chemical composition, type, and quantity can impact the level of wear experienced by restorations. Decreasing the filler content enhances the polishability but diminishes the overall resistance to wear^37^.

In this study, patient acceptability was assessed, with patient satisfaction recognized as a crucial factor contributing to the success of dental treatment. The results showed that all patients demonstrated a satisfactory level of acceptability, as indicated by the Alpha score. This is supported by Nagi et al., who reported a high level of patient satisfaction with the monoshade universal RC^38^.

One of the primary constraints of this research is that a 12-month period might be insufficient to identify substantial changes. Therefore, prolonged clinical assessment studies may provide a more valuable evaluation of the clinical performance of monoshade universal RCs.

Further in-vivo investigations are required to explore the impact of food and beverage stains on the long-term shade-matching permanence of Omnichroma dental resin restorative composite. Additionally, assessing the influence of pH variations and the effects of acids on the microstructure of Omnichroma composite is crucial, as it allows for correlation with its mechanical and esthetic properties. Further evaluation of contemporary monoshade universal resin composites and the possible differences between them may be useful for assessing the clinical success of these materials.

## Conclusion

Based on the results of this clinical trial; Omnichroma demonstrated comparable performance to Tetric-N-Ceram and can be utilized as a substitute for polyshade nanohybrid composite when chair side time is an essential concern. Class-I and II carious lesions are successfully treated with monoshade composites, which exhibit good color matching and clinical performance.

## Data Availability

The corresponding author can provide the datasets utilized and/or examined in the present study upon a reasonable request.
